# Dibutyryl-cAMP attenuates pulmonary fibrosis by blocking myofibroblast differentiation via PKA/CREB/CBP signaling in rats with silicosis

**DOI:** 10.1186/s12931-017-0523-z

**Published:** 2017-02-21

**Authors:** Yan Liu, Hong Xu, Yucong Geng, Dingjie Xu, Lijuan Zhang, Yi Yang, Zhongqiu Wei, Bonan Zhang, Shifeng Li, Xuemin Gao, Ruimin Wang, Xianghong Zhang, Darrell Brann, Fang Yang

**Affiliations:** 1 Basic Medical College, Hebei Medical University, No. 361 Zhongshan Road, Shijiazhuang city, Hebei province China; 2 Medical Research Center, North China University of Science and Technology, Tangshan, Hebei 063009 China; 3 Traditional Chinese Medicine College, North China University of Science and Technology, Tangshan, Hebei 063009 China; 4 Basic Medical College, North China University of Science and Technology, Tangshan, Hebei 063009 China; 5 Department of Neuroscience and Regenerative Medicine, Medical College of Georgia, Augusta University, Augusta, GA 30912 USA

**Keywords:** Silicosis, Myofibroblast, CAMP, PKA, CREB, Smad

## Abstract

**Background:**

Myofibroblasts play a major role in the synthesis of extracellular matrix (ECM) and the stimulation of these cells is thought to play an important role in the development of silicosis. The present study was undertaken to investigate the anti-fibrotic effects of dibutyryl-cAMP (db-cAMP) on rats induced by silica.

**Methods:**

A HOPE MED 8050 exposure control apparatus was used to create the silicosis model. Rats were randomly divided into 4 groups: 1)controls for 16 w; 2)silicosis for 16 w; 3)db-cAMP pre-treatment; 4) db-cAMP post-treatment. Rat pulmonary fibroblasts were cultured in vitro and divided into 4 groups as follows: 1) controls; 2) 10^−7^mol/L angiotensin II (Ang II); 3) Ang II +10^−4^ mol/L db-cAMP; and 4) Ang II + db-cAMP+ 10^−6^ mol/L H89. Hematoxylin-eosin (HE), Van Gieson staining and immunohistochemistry (IHC) were performed to observe the histomorphology of lung tissue. The levels of cAMP were detected by enzyme immunoassay. Double-labeling for α-SMA with Gαi3, protein kinase A (PKA), phosphorylated cAMP-response element-binding protein (p-CREB), and p-Smad2/3 was identified by immunofluorescence staining. Protein levels were detected by Western blot analysis. The interaction between CREB-binding protein (CBP) and Smad2/3 and p-CREB were measured by co-immunoprecipitation (Co-IP).

**Results:**

Db-cAMP treatment reduced the number and size of silicosis nodules, inhibited myofibroblast differentiation, and extracellular matrix deposition in vitro and in vivo. In addition, db-cAMP regulated Gαs protein and inhibited expression of Gαi protein, which increased endogenous cAMP. Db-cAMP increased phosphorylated cAMP-response element-binding protein (p-CREB) via protein kinase A (PKA) signaling, and decreased nuclear p-Smad2/3 binding with CREB binding protein (CBP), which reduced activation of p-Smads in fibroblasts induced by Ang II.

**Conclusions:**

This study showed an anti-silicotic effect of db-cAMP that was mediated via PKA/p-CREB/CBP signaling. Furthermore, the findings offer novel insight into the potential use of cAMP signaling for therapeutic strategies to treat silicosis.

**Electronic supplementary material:**

The online version of this article (doi:10.1186/s12931-017-0523-z) contains supplementary material, which is available to authorized users.

## Background

Silicosis is a fibrotic disease caused by inhalation of crystalline silica dust and the subsequent formation of silicotic lesions and extracellular matrix (ECM) deposition by activated myofibroblasts [1–3]. Myofibroblasts are α-smooth muscle actin (α-SMA)-expressing cells that secrete ECM components and originate from diverse sources that depend on physiological stimuli [4]. Ang II, a major renin-angiotensin peptide can increase expression of transforming growth factor-β (TGF-β) and promote an Ang II/TGF-β1 “autocrine loop,” which initiates a fibrogenic signaling pathway [5]. Accumulating evidence suggests that TGF-β/Smad signaling is a mediator of pro-fibrotic effects of Ang II and promotes myofibroblast differentiation [6]. Ang II has been suggested to be involved in lung inflammation via release of pro-inflammatory cytokines [7], which induce alveolar epithelial cell apoptosis [8]. Additional studies have shown that Ang II is up-regulated in serum and lung tissue in a silicosis rat model [3]. Furthermore, treatment with ACE inhibitors and Ang II receptor blockers have been shown to improve pulmonary fibrosis [9, 10]. Collectively, these findings suggest that Ang II signaling has a critical role in the pathogenesis of lung fibrosis.

In previous work, a preliminary proteomic profile analysis indicated that cAMP signaling might have anti-silicotic effects [11]. cAMP is generated by adenylyl cyclase (AC) in response to activation of stimulatory G protein (Gs) or by blocking inhibitory G protein (Gi), and it is degraded by phosphodiesterase (PDE). Increases in cAMP inhibit fibroblast proliferation and ECM synthesis, which have anti-fibrotic effects in vitro and in vivo [4, 12]. A PDE inhibitor (roflumilast) [13], an AC activator (forskolin) [14], or an exogenous prostaglandin E2, such as aminophylline, have been shown to have anti-fibrotic effects as well [15]. In addition, cAMP controls inhibition of fibroblast activation and myofibroblast transition. Studies suggest that increasing concentrations of cAMP not only prevent cardiac fibroblast-to-myofibroblast transformation, but also reverse the pro-fibrotic myofibroblastic phenotype [14, 16]. Furthermore, over-expression of PDE2 in cardiac fibroblasts reduced basal and isoprenaline-induced cAMP synthesis, and this effect was sufficient to induce fibroblast-to-myofibroblast conversions even without exogenous pro-fibrotic stimuli [17].

Dibutyryl-cAMP (db-cAMP) is a cell permeable analogue of cAMP that can prevent acute pulmonary vascular injury induced by endotoxin [18]. It has also been shown to attenuate ischaemia/reperfusion injury in rat lungs [19], and inhibit fibroblast proliferation and collagen production [20, 21]. PKA, the classical cAMP effector, can phosphorylate cAMP-response element-binding protein (CREB) at serine 133, and as such is associated with co-activation of the CREB binding protein (CBP) and transactivation of cAMP-responsive genes [22–25]. Increased cAMP levels has been shown to abolish TGF-β1-induced interaction of Smad3 with CBP, and to decrease ECM [22, 24]. However, how db-cAMP/PKA/CREB/CBP signaling attenuates silicosis is unknown.

Here, we investigated the anti-fibrotic effect of db-cAMP in a silicosis rat model and in myofibroblasts induced by Ang II, and studied the regulatory effect of db-cAMP upon Gαs and Gαi. We also examined the ability of db-cAMP to regulate the interaction of CBP with Smad2/3 through PKA/CREB signaling. The results of the studies implicate an important role for cAMP signaling in silicosis, which could lead to development novel therapies for treatment of silicosis.

## Methods

### Animal Experiments

All animal experiments were approved by the North China University of Science and Technology Institutional Animal Care and Use Committees (2013-038). Male Wistar rats (3 weeks-of-age) were from Vital River Laboratory Animal Technology Co. Ltd. (SCXY 2009-0004, Beijing, China). A HOPE MED 8050 exposure control apparatus (HOPE Industry and Trade Co. Ltd, Tianjin, China) was used to create the silicosis model (Additional file 1: Figure S1). This system can be set to a certain dust concentration and it is a non-invasive instrument for allowing animal inhalation. Settings were as follows: exposure chamber volume 0.3 m^3^, cabinet temperature 20–25 ^o^C, humidity 70–75%, pressure -50 to + 50 Pa, oxygen concentration 20%, flow rate of SiO_2_ (5 um silica particles, s5631, Sigma-Aldrich) 3.0–3.5 ml/min, dust mass concentration in the cabinet 2000 mg/m^3^, and each animal inhaled for 3 h per day. db-cAMP (10 mg/kg/d) or 0.9% saline was given by subcutaneous injection.

A preliminary experiment showed that cellular lesions are observed in rats exposed to silica for 4 w, and confluent multi-nodular or diffuse distribution of cellular lesions is found in rats exposed to silica for 16 w (Additional file 2: Figure S2). Based on the results of the preliminary experiment, rats were randomly divided into 4 groups: 1)controls for 16 w (treated with 0.9% saline for 16 w); 2)silicosis for 16 w (treated with 0.9% saline 48 h before SiO_2_ inhaling, and then continued treatment for 16 w); 3)db-cAMP pre-treatment (treated with db-cAMP 48 h before inhaling of SiO_2_, and then continued for 16 w); 4) db-cAMP post-treatment (inhaling of SiO_2_ and treated with 0.9% saline for 4 w and db-cAMP for another 12 w). Silicotic rats treated with or without db-cAMP were all exposed to silica for 16 weeks.

### Cell culture

Lung fibroblasts were isolated from minced tissue and plated on 25 cm^2^ plates in DMEM (BI-SH0019, BI, Kibbutz Beit-Haemek, Israel) medium containing 10% FBS (10099141, Gibco, Thermo Fisher Scientific) and 1% penicillin-streptomycin. Cells were cultured in a humidified atmosphere of 5% CO_2_ and 95% air at 37 ^o^C. Cells at 80% confluence were cultured in FBS-free DMEM medium for 24 h, when most cells were quiescent. Next, cells were divided into four groups and were cultured for 24 h as follows: 1) controls; 2) 10^−7^mol/L Ang II (A9525, Sigma-Aldrich); 3) Ang II +10^−4^ mol/L db-cAMP: db-cAMP treatment was started 1 h before Ang II stimulation; and 4) Ang II + db-cAMP+ 10^−6^ mol/L H89 (10010556, Cayman): H89 treatment was started 1 h before db-cAMP treatment.

### Histological analysis

The right lower lungs were fixed in 4% paraformaldehyde, paraffin embedded, and then sectioned for pathophysiological observation. Lung tissue slides were stained with hematoxylin-eosin (HE) to assess fibrosis. Van Gieson (VG) staining was used to measure collagen fiber deposition. The number and size/area of silicosis nodules were counted by CellSense software and Olympus DP80 system. Based on the VG staining, the area of collagen deposition ≥50% in a silicotic nodule was defined as a score of “2”, and an area <50% was defined as a score of “1”. The silicotic area (product of area and collagen score) and the number of silicotic nodules were homogenized by the total area of lung section.

### Immunohistochemistry (IHC)

Paraffin-embedded sections of lung tissue were assessed with IHC. Endogenous peroxidases were quenched with 0.3% H_2_O_2_, and antigen retrieval was performed using a high-pressure method on dewaxed tissue sections. Samples were then incubated with primary antibodies against α-SMA (ab32575, Eptomics, Burlingame, CA) and p-CREB (ab32096, Abcam) overnight at 4 °C, followed by incubation with a secondary antibody (PV-6000, Beijing Zhongshan Jinqiao Biotechnology Co. Ltd, China) at 37 °C for 20 min. Immunoreactivity was visualized with DAB (ZLI-9018, ZSGB-BIO, Beijing, China). Brown staining was considered positive.

### Immunofluorescence

Co-staining was performed on lung tissue sections and fibroblasts. Samples were incubated in 10% donkey serum (92590, Temecula, CA) for 30 min at 37 °C. After co-incubation overnight at 4 °C with Gαi3 (sc-365422, Santa Cruz Biotechnology, Dallas, TX)/α-SMA, PKA(ADI-KAS-PK017, Enzo, Farmingdale, NY)/α-SMA, p-CREB/α-SMA and p-Smad2/3 (ART1568, Antibody Revolution, San Diego, CA)/α-SMA, sections were incubated with products from Novex (Life Technologies, Frederick, MD): donkey anti-rabbit IgG (H + L) FITC (A16024), donkey anti-mouse IgG (H + L) TRITC (A16016), Alexa Fluor 647 donkey anti-goat IgG (H + L) (A21447) or donkey anti-rabbit IgG (H + L) TRITC (A16028) and donkey anti-mouse IgG (H + L) FITC (A16018) for 60 min each at 37 °C in blocking buffer. Nuclei were stained with DAPI (14285, Cayman, Ann Arbor, MI) for 5 min. Cells or tissues were visualized under an Olympus DP80 microscope and were analyzed with image software (Cell Sens 1.8, Olympus Corporation, Germany).

### Western blot

The lung tissue or cells were lysed in RIPA buffer (1% NP-40, 0.5% sodium deoxycholate, 0.1% SDS, 150 mM NaCl, 1 mM EDTA, and 50 mM Tris-HCl, pH 7.5) containing a protease inhibitor cocktail (P2714-1BTL, Sigma-Aldrich, St. Louis, MO). Protein concentrations in supernatants were measured with a Bradford assay (PC0020, Solarbio, Beijing, China). Protein samples (20 μg/lane) were separated with 10% SDS-PAGE and electrophoretically transferred to PVDF membranes. The membranes were then blocked with Tris-buffered solution with 0.1% Tween supplemented with 5% fat-free milk, and incubated overnight at 4 ^o^C with primary antibody against collagen type I (Col I) (ab34710, Abcam, Cambridge, UK), Fibronectin (Fn) (ab45688, Eptomics, Burlingame, CA), α-SMA, Gαs (sc-135914, Santa Cruz Biotechnology, Dallas, Texas), Gαi2 (sc-7276, Santa Cruz Biotechnology, Dallas, TX), Gαi3, PKA, p-CREB, CREB (ab32515, Abcam, Cambridge, UK), p-Smad2/3, total-Smad2/3 (3308791, BD Biosciences, San Jose, CA) or CBP (ab2832, Abcam, Cambridge, UK). The membranes were then probed with a peroxidase-labeled affinity-purified antibody to rabbit/mouse IgG (H + L) (074–1506/074–1806, Kirkegard and Perry Laboratories, Gaithersburg, MD) and peroxidase-labeled affinity-purified antibody to goat IgG (H + L) (14–13-06, Kirkegard and Perry Laboratories, Gaithersburg, MD). Target bands were visualized by the addition of ECL^TM^ Prime Western Blotting Detection Reagent (RPN2232, GE Healthcare, Hong Kong, China). Results were normalized with β-action (sc-47778, Santa Cruz Biotechnology) or GAPDH (sc-25778, Santa Cruz Biotechnology).

### Co-immunoprecipitation (Co-IP)

For performance of Co-IP, lung fibroblast cells were lysed with RIPA buffer and centrifuged at 13,000 × g for 10 min at 4 ^o^C. The supernatants were collected, and immunoprecipitation was performed with an antibody to CBP, and immune complexes were captured using ProteinA/G-agarose beads (SC-2003, Santa Cruz Biotechnology), according to the manufacturer’s instructions. Protein was eluted by boiling in 1× concentrated sample buffer and analyzed by Western blot.

### Enzyme immunoassay (EIA)

The levels of cAMP in cellular and lung tissue were determined by using a cAMP EIA kit (581001, Cayman, Ann Arbor, MI, USA), according to the manufacturer’s instruction. Each assay point was performed in triplicate. The content of cAMP was calculated according to the standard curve.

### Statistical analysis

Data are presented as means ± SEM. Comparisons between multiple independent groups were performed with one-way ANOVA, followed by a *post* hoc analysis with the Bonferroni test using SPSS13.0 software. Group differences with *p*-values < 0.05 indicate a statistically significant difference.

## Results

### Db-cAMP reduced expression of ECM and myofibroblast differentiation in rats exposed to silica and in fibroblasts induced by Ang II

**Fig. 1:**
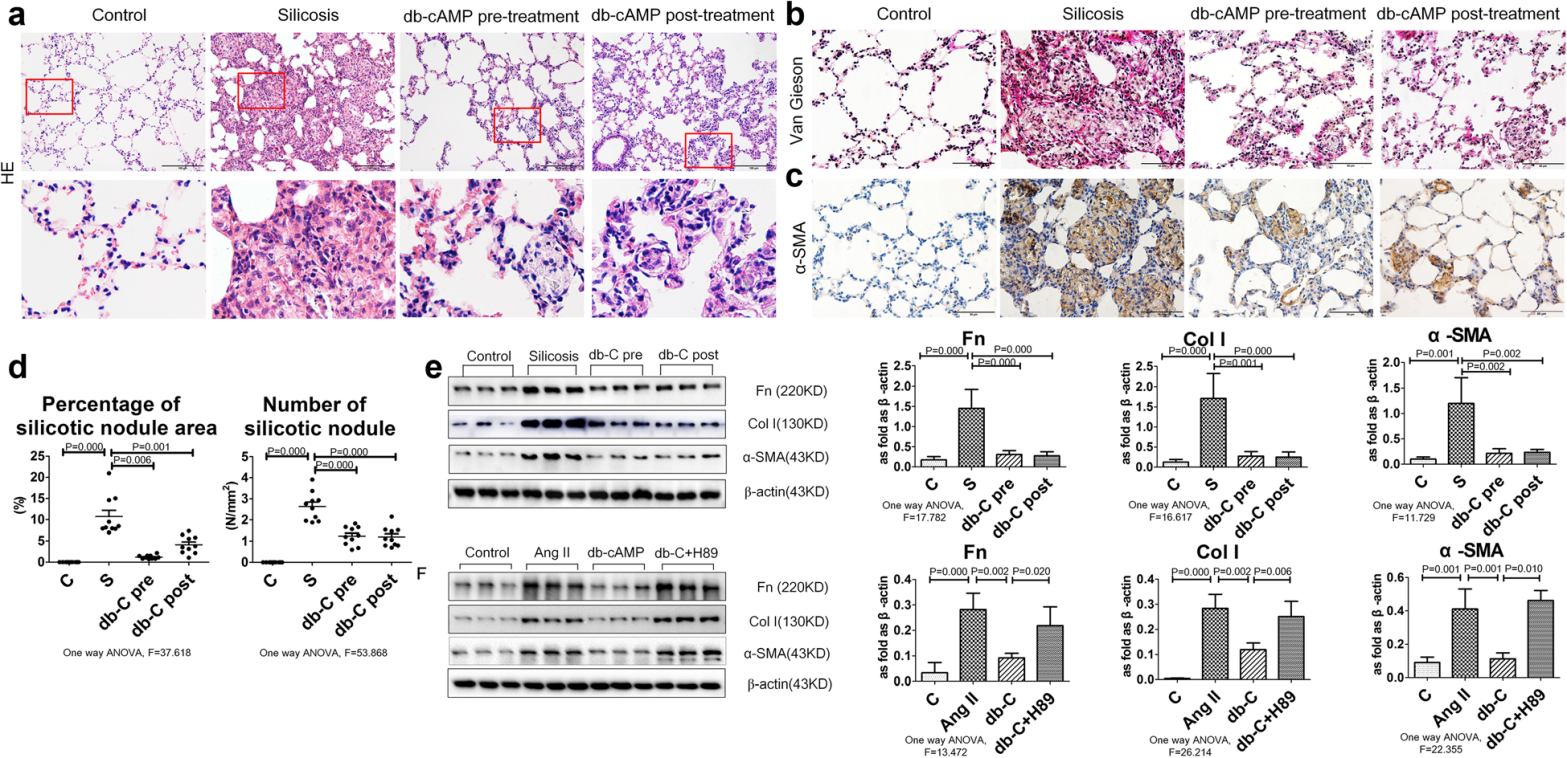
Myofibroblast differentiation and ECM deposition is regulated by db-cAMP. **a** Lung tissue stained with HE staining; **b** Lung tissue stained with Van Gieson staining; **c** Immunohistochemical measurement of α-SMA expression in rat lung silicosis tissue; **d** Percentage of silicotic nodule area and number of silicotic nodule; **e** Fn, Col I and α-SMA expression in lung tissue (Western blot). Data are means ± SEM; *n* = 6 independent experiments; **f** Fn, Col I and α-SMA expression in fibroblasts (Western blot). Data are means ± SEM; *n* = 6 independent experiments

HE and Van Gieson staining (Fig. 1a, b and d) revealed that db-cAMP pre- and post-treatment reduced the number and size of silicotic nodules, as well as the accumulation of collagenous fibers. IHC staining of tissue indicated positive expression of α-SMA was marked in myofibroblasts, which were surrounded by macrophages and unevenly distributed in the interstitial fibrotic area (Fig. 1c). In addition, Western blot analysis demonstrated that Fn, Col I and α-SMA expression were increased in the silica inhalation for 16 W group, as compared with controls (Fig. 1e). Intriguingly, pre-treatment with db-cAMP reduced these fibrotic changes, and db-cAMP post-treatment had the same effect.

After Ang II induction, the synthesis of Fn, Col I and α-SMA were significantly increased in cultured lung fibroblasts, as compared to controls (Fig. 1f). In contrast, pretreatment with db-cAMP reduced Fn, Col I and α-SMA expression. Specifically blocking the PKA signal by H89 reduced the effect of db-cAMP on Ang II.

### Db-cAMP regulated Gαs/Gαi, cAMP contents in silicosis and in myofibroblasts induced by Ang II

**Fig. 2:**
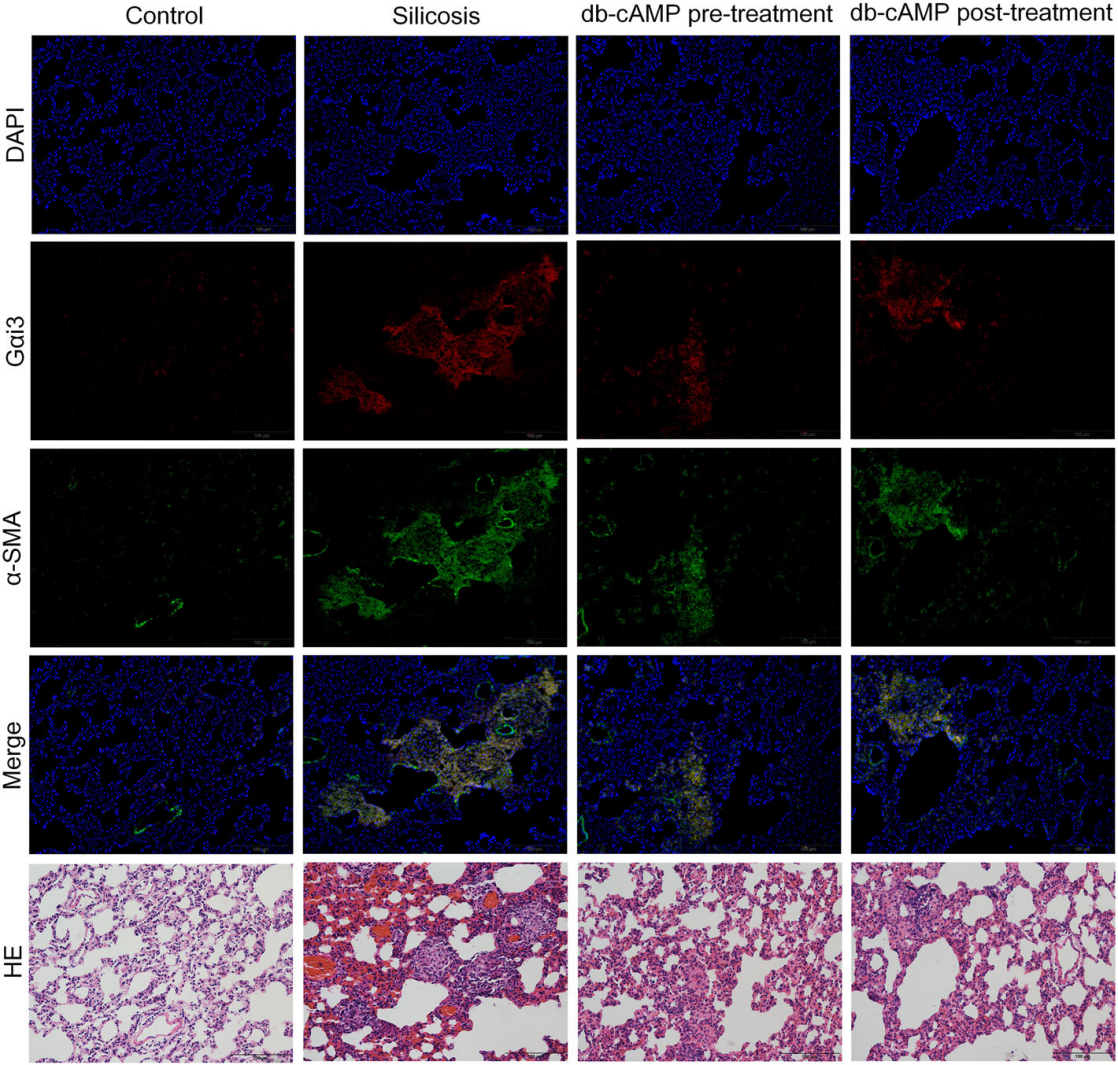
The Co-expression of Gαi3 and α-SMA in rat silicosis lung tissue is regulated by db-cAMP (immunofluorescence)

**Fig. 3:**
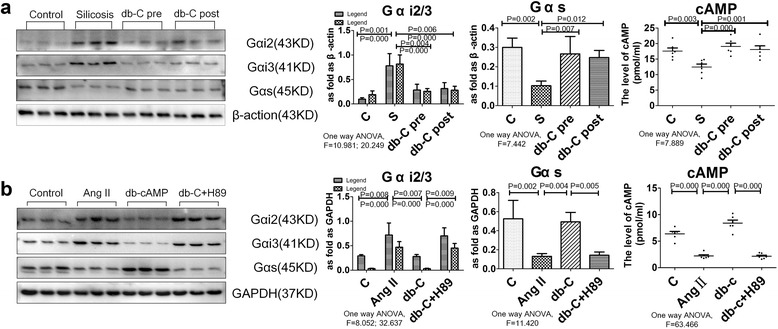
The expression of Gαs/Gαi protein and endogenous cAMP level is regulated by db-cAMP. **a** Gαs, Gαi2, Gαi3 and cAMP expression in rat lung tissue (Western blot, EIA); Data are means ± SEM; *n* = 6 independent experiments; **b** Gαs, Gαi2, Gαi3 and cAMP expression in fibroblasts (Western blot, EIA). Data are means ± SEM; *n* = 6 independent experiments

As shown in Fig. 2, co-expression of Gαi3 and α-SMA were increased significantly in silicotic nodules and interstitial fibrotic regions, as compared to controls. In the area of interstitial fibrosis or alveolar wall broadening, there was significant Gαi3 protein positive expression. Pre- or post-treatment with db-cAMP decreased expression of both Gαi3 and α-SMA. As shown in Fig. 3a, Western blot analysis confirmed that db-cAMP pre- or post-treatment decreased the expression of Gαi2 and Gαi3 in silicotic lung tissue, while up-regulating Gαs, cAMP.

To investigate whether Gαs/Gαi proteins were involved in myofibroblast differentiation in vitro, we quantified expression of Gαs, Gαi2, and Gαi3 in Ang II-treated lung fibroblasts. As shown in Fig. 3b, Western blot analysis demonstrated that Ang II treatment significantly reduced Gαs, while enhancing expression of Gαi2 and Gαi3. Furthermore, pre-treatment with db-cAMP increased Gαs and reduced the up-regulation of Gαi2 and Gαi3 induced by Ang II. Correspondingly, the level of cAMP in fibroblasts was significantly increased. Finally, all of the effects of db-cAMP were inhibited by the PKA signaling inhibitor, H89 (Fig. 3b).

### Db-cAMP inhibited myofibroblast differentiation by promoting nuclear translocation of p-CREB via PKA signaling

**Fig. 4:**
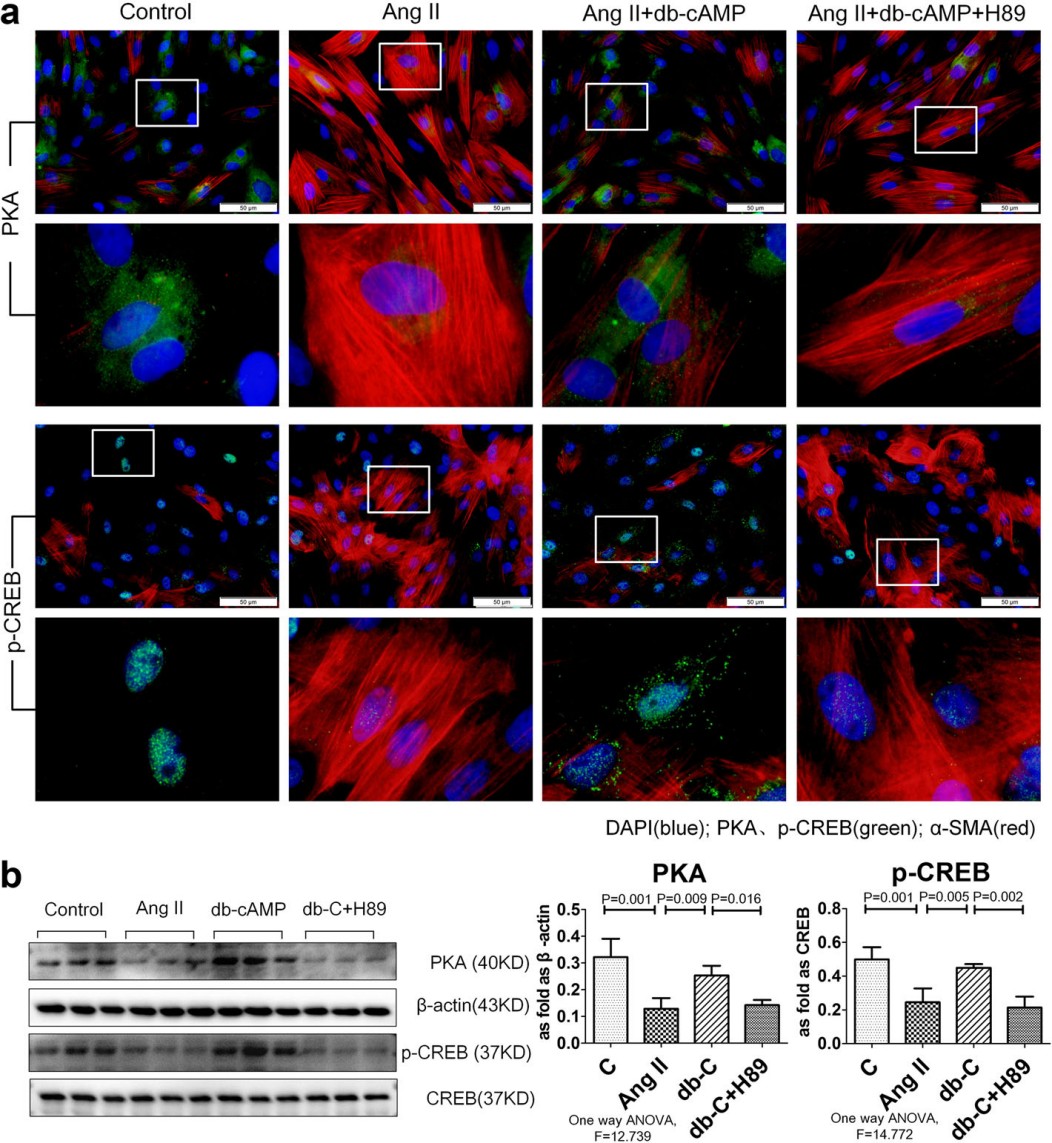
Activation of db-cAMP on PKA/p-CREB signaling in vitro. **a** Co-expression of PKA/α-SMA and p-CREB/α-SMA in fibroblasts (immunofluorescence; *red*: α-SMA; *green*: PKA and p-CREB; *blue*: DAPI); **b** PKA and p-CREB expression in fibroblasts (Western blot); Data are means ± SEM; *n* = 6 independent experiments

Since PKA is a classic cAMP effector, we next investigated whether myofibroblast differentiation could be inhibited by PKA/CREB signaling. As shown in Fig. 4a, immunofluorescent staining revealed that PKA and p-CREB were significantly decreased in Ang II-induced fibroblasts, and this effect was accompanied by up-regulation of α-SMA in the cytoplasm, as compared with controls. Furthermore, positive expression of p-CREB was observed in nuclei after fibroblast treatment with db-cAMP, and decreased in the Ang II group or H89 treatment group. In line with the immunofluorescent data, Western blot results confirmed that db-cAMP treatment inhibited the Ang II-induced down-regulation of PKA and p-CREB, and this effect was reversed with by H89 (Fig. 4b).

**Fig. 5:**
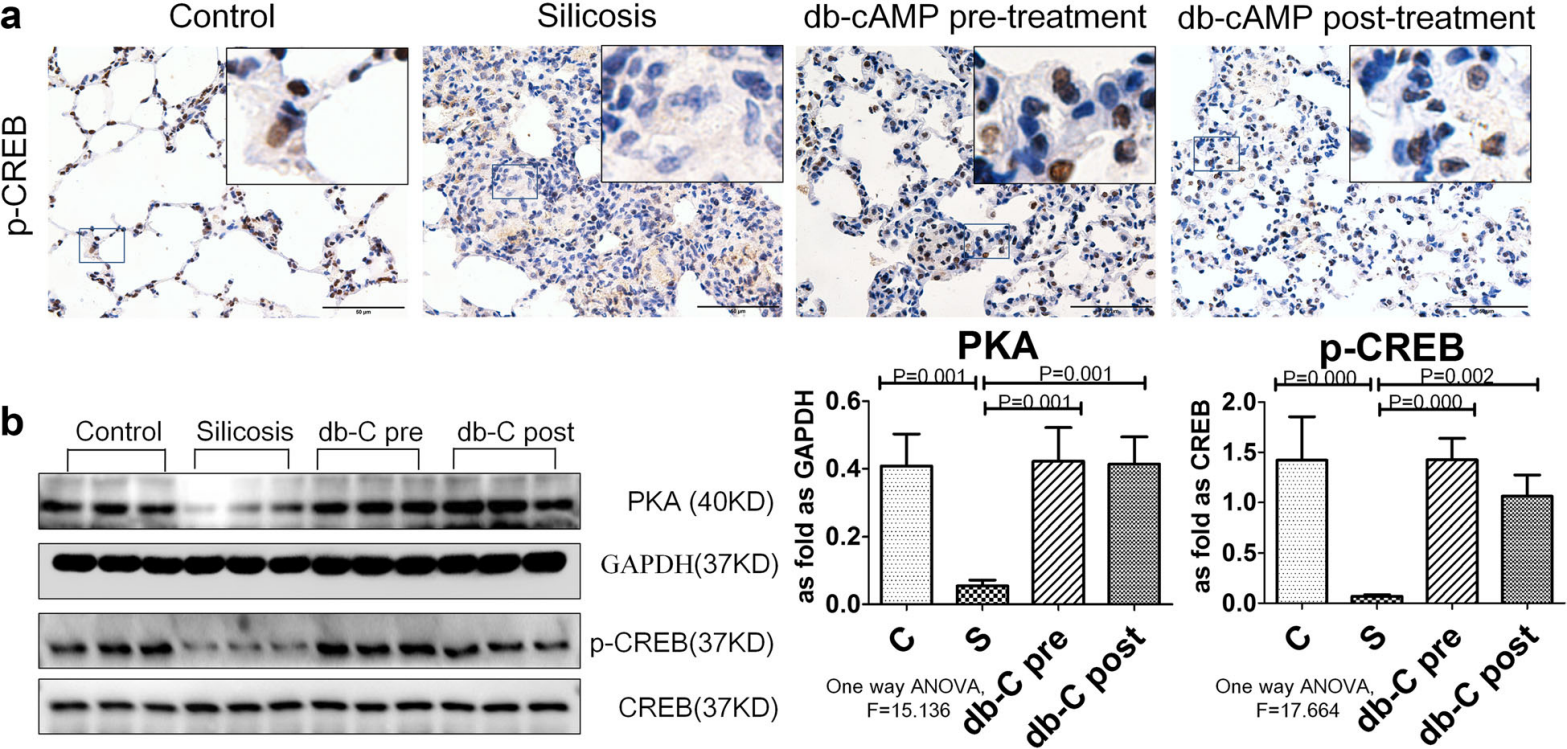
The expression of PKA/p-CREB protein is regulated by db-cAMP in vivo. **a** p-CREB expression in silicotic rat lung tissue (IHC); **b** PKA and p-CREB expression in rat silicotic lung tissue (Western blot); Data are means ± SEM; *n* = 6 independent experiments

We next measured expression and localization of p-CREB in the silicosis model using IHC staining. Positive expression of p-CREB was observed in the nucleus of normal lung tissue, with no staining observed in silicotic nodules (Fig. 5a). Furthermore, Western blot results showed that the levels of PKA and p-CREB were significantly reduced in the silicosis group (Fig. 5b), while pre- or post-treatment with db-cAMP promoted expression of both PKA and p-CREB.

### Db-cAMP inhibition of myofibroblast differentiation is dependent upon p-CREB/CBP signaling interference with Smad2/3 signaling

**Fig. 6:**
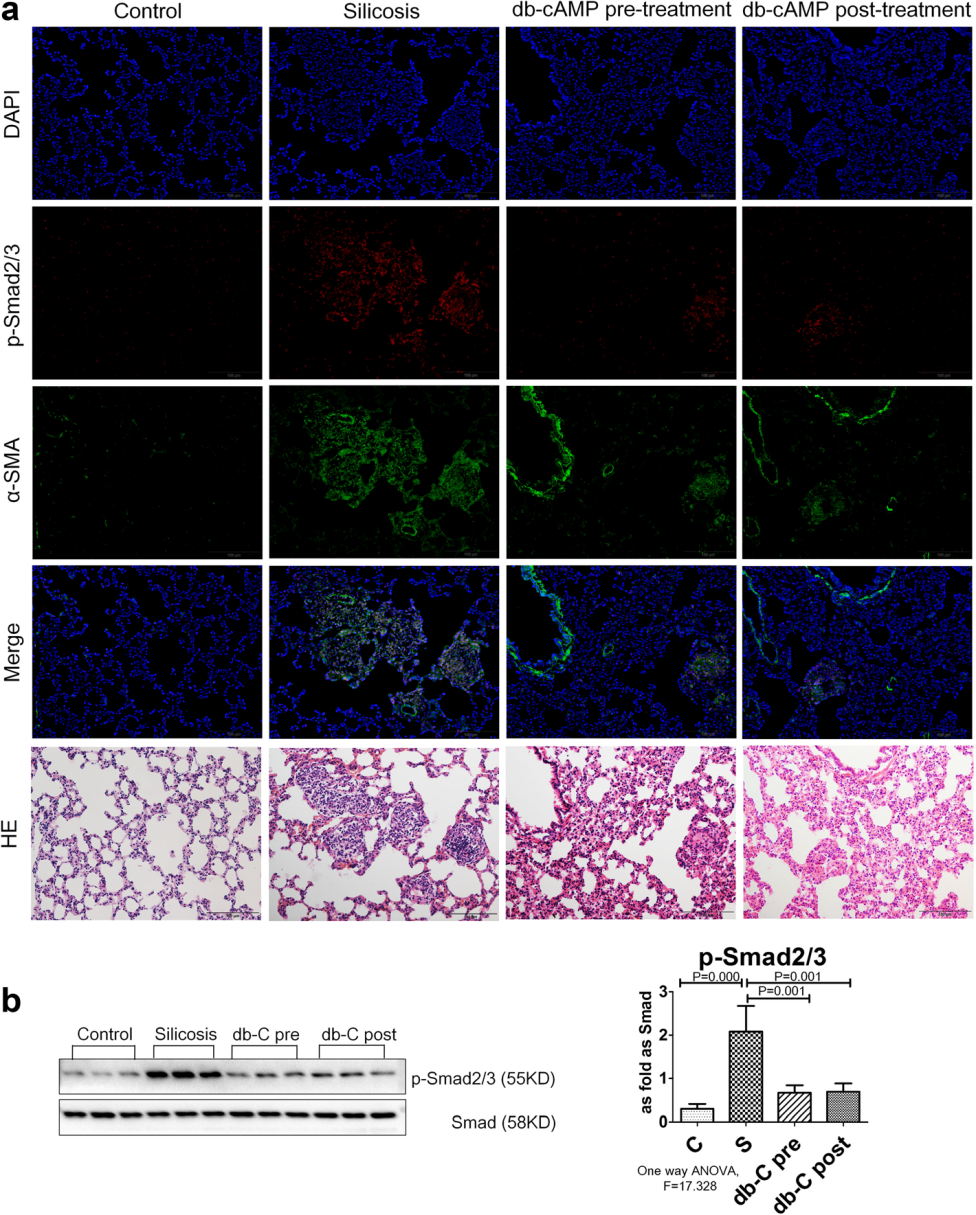
The expression of p-Smad2/3 protein is regulated by db-cAMP in vivo. **a** Co-expression of p-Smads/α-SMA in lung tissue (immunofluorescence; *red*: p-Smad2/3; *green*: α-SMA; *blue*: DAPI); **b**The expression of p-Smad2/3 and Smad2/3 in vivo (Western blot); Data are means ± SEM; *n* = 6 independent experiments

**Fig. 7:**
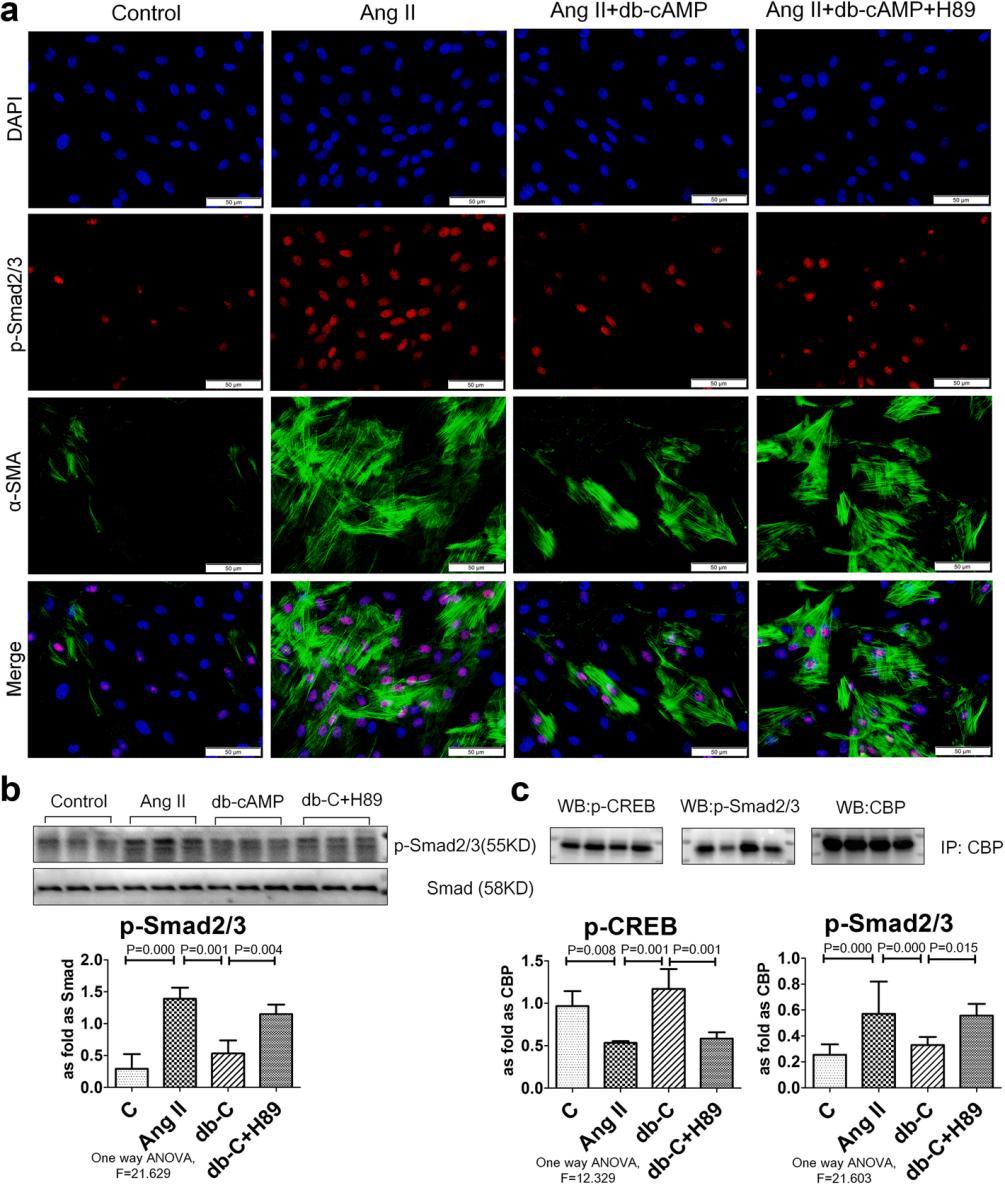
Interaction of p-CREB and p-Smad2/3 binding with CBP is regulated by db-cAMP. **a** Co-expression of p-Smads/α-SMA in fibroblasts (immunofluorescence; *red*: p-Smad2/3; *green*: α-SMA; *blue*: DAPI); **b** The expression of p-Smad2/3 in vitro (Western blot); Data are means ± SEM; *n* = 6 independent experiments; **c** p-CREB and p-Smad2/3 binding with CBP measured by co-IP, Data are means ± SEM, *n* = 3 independent experiments

Smad2/3 is a major pro-fibrotic signaling molecule that can activate α-SMA promoter activity and promote myofibroblast differentiation. Examination of p-Smad2/3 by immunofluorescent staining and Western blot analysis showed that it was significantly increased in the silicotic rat model and in fibroblasts induced with Ang II (Figs. 6 and 7a and b). Treatment with db-cAMP inhibited up-regulation of p-Smad2/3 in vivo and in vitro. Blocking PKA signaling by H89 prevented inhibition of db-cAMP in Ang II-induced myofibroblasts. With Co-IP analysis (Fig. 7c), we noted an interaction of CBP with p-CREB or p-Smad2/3. Co-IP data from fibroblast lysates with anti-CBP antibodies indicated increased expression of p-Smad2/3, and down-regulation of p-CREB in fibroblasts induced with Ang II. Treatment with db-cAMP promoted association of p-CREB and inhibited association of p-Smad2/3 with CBP. Thus, p-CREB/CBP interactions inhibited binding of p-Smad2/3 to CBP and inhibited p-Smad2/3 nuclear translocation.

## Discussions

Over the past decade, tracheal instillation of silica dust has been extensively used as a silicosis model to reveal the possible mechanism of the occurrence and development of silicosis [3, 26, 27]. In the current study, our rat model was created using silica that was inhaled from a HOPE MED8050 exposure control apparatus, which allows greater control and more closely approximates exposure and development of silicosis in humans. After inhalation of SiO_2_ for 4 w, silicotic nodules were visible in lung tissue and these increased by 8 w. Fibrous and cellular silicotic nodules with diffuse interstitial fibrosis were observed in rats at 16 w. Based on the these results, inhalation of SiO_2_ for 16 w was used for further evaluation of the anti-fibrotic effects of db-cAMP. Further characterization with IHC revealed that α-SMA-positive expressing myofibroblasts surrounded macrophages and were irregularly distributed in interstitial fibrotic areas, further confirming the robustness of the silicosis model. It is well known that RAS is a key mediator of lung fibrosis pathogenesis and that Ang II potently induces fibrosis [3, 28, 29]. In agreement, treatment of fibroblasts with Ang II in our study markedly increased expression of Fn, Col I and α-SMA. Thus, the rat silicosis model used in our study was characterized by robust ECM deposition and myofibroblast differentiation, which was mediated at least in part, by RAS signaling activation.

Increase in cAMP has been previously shown to inhibit fibroblast proliferation and ECM synthesis, and to be correlated with anti-fibrotic effects in vitro and in vivo [4, 12]. Furthermore, cAMP was previously shown to protect against pulmonary fibrosis induced by bleomycin, chronic obstructive pulmonary disease, and experimental acute lung injury [30–32]. In the current study, the results showed that treatment with db-cAMP reduced the number and size of silicotic nodules and collagenous fibers, and inhibited ECM synthesis and myofibroblast differentiation in vitro and in vivo. Also, db-cAMP promoted expression of Gαs protein and inhibited expression of Gαi protein, which increased endogenous cAMP. From a functional standpoint, previous work has shown that Gαi2 and Gαi3 can contribute to redundant and overlapping inflammation in an experimental model of immune complex-induced inflammation [33]. Furthermore, Gαi2-deficient mice had less recruitment of macrophages in lipopolysaccharide-induced lung injury, and decreased RAW 264.7 cell migration and motility [34]. In contrast, Gαs has been shown to be required for adenosine-induced barrier enhancement effects in human pulmonary artery endothelial cells [35]. Thus, the balance of Gαs/Gαi in lung fibrosis may regulate cAMP, ECM, myofibroblast differentiation, inflammation and endothelial cell barrier function.

Mechanistically, our study demonstrated a dramatic down-regulation of cAMP/PKA/p-CREB signaling in the silicosis model and in induced fibroblasts, and this effect was significantly reduced with db-cAMP treatment. Furthermore, the PKA inhibitor H89 prevented the anti-fibrotic effects of db-cAMP. These findings suggest that regulation of cAMP/PKA/p-CREB signaling can have important anti-fibrotic effects in silicosis. In support of this possibility, another study found that the antitussive drug, nosacpine stimulated a rapid and profound activation of PKA in a pulmonary fibrosis model, which correlated with significant anti-fibrotic effects in vitro and in vivo [36]. In another study, *Prkar1a* null primary mouse embryonic fibroblasts, which display constitutive PKA signaling, had down-regulated vimentin and α-SMA accompanied with up-regulation of E-cadherin, suggesting that activation of PKA signaling promoted mesenchymal to epithelial transition [37].

**Fig. 8:**
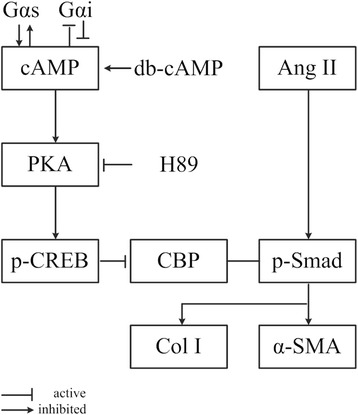
Proposed Mechanism of cAMP Anti-Fibrotic Effects. Anti-fibrotic effects of cAMP are proposed to involve 1) increased PKA and p-CREB, 2) down-regulation of p-Smad2/3 binding to CBP, 3) concomitant reduced activation of p-Smads and the Smad-induced genes, Collagen I, Fn and α-SMA in induced fibroblasts, and 4) a resultant inhibition of myofibroblast differentiation

Accumulating evidence indicates that Smad2/3 is extensively activated in fibrotic disease and in animal experiments, regulating various genes including α-SMA and Col I [38, 39]. Previous studies confirm that Ang II is critical to pathological organ remodeling via activating Smad signaling to cause pro-fibrotic effects by promoting myofibroblast differentiation and excessive synthesis and deposition of ECM [40–44]. Herein, we observed that nuclear expression of p-Smad2/3 in vitro and in vivo was related to myofibroblast differentiation and ECM synthesis, which was reduced by db-cAMP via PKA signaling. CREB is a well-known transcription factor of the basic leucine zipper family and upon activation it promotes interactions with co-activators such as CBP, E1A binding protein p300 (P300), and CREB-regulated transcription co-activator 2 (CRTC) by adapting DNA-binding and transcriptional activation [45, 46]. Interestingly, CBP is required for a multi-protein complex among p-Smad3, β-catenin and CBP at the promoter to regulate α-SMA expression in RLE-6TN cells treated with TGF-β1 [23]. Moreover, increasing intracellular cAMP levels can phosphorylate CREB, and recruiting CBP in the nucleus from Smad proteins inhibits the effects of TGF-β1/Ang II on fibroblasts [22, 24, 47]. In our study, Co-IP showed that db-cAMP increased p-CREB, while down-regulating p-Smad2/3 binding to CBP, which reduced activation of p-Smads in induced fibroblasts. IHC data further showed that positive nuclear expression of p-CREB occurred chiefly in normal lung tissue, and expression was lost in silicotic nodules. In contrast, positive expression of p-smad2/3 was mainly located in silicotic nodules. p-CREB location suggested that it might appear in multiple cell types and regulate an anti-fibrotic process. Thus, the results of our study provides evidence that cAMP has anti-fibrotic effects in vitro and in vivo, and that these effects depend on PKA/p-CREB signaling by disturbing p-Smad2/3 binding with CBP, and inhibiting myofibroblast differentiation in a model of silicosis (Fig. 8).

## Conclusions

Taken together, in the present study, we provide evidence that db-cAMP has anti-fibrotic effects in vitro and in vivo. The effects were dependent on PKA/p-CREB signaling to disrupt p-Smad2/3 binding with CBP, and ultimately result in inhibition of myofibroblast differentiation in silicosis.

## Additional files

Additional file 1: Figure S1. The HOPE-MED 8050 exposure control apparatus. A) barrier system; B) silica inhalation system; C) waste disposal system (JPG 2785 kb)
Additional file 2: Figure S2. The morphological observation of rat exposed to silica. (JPG 4540 kb)

## Abbreviations

AC: Adenylyl cyclase
Ang II: Angiotensin II
CBP: CREB binding protein
Co-IP: Co-Immunoprecipitation
db-cAMP: Dibutyryl-cAMP
ECM: Extracellular matrix
EIA: Enzyme Immunoassay
HE: Hematoxylin-eosin
IHC: Immunohistochemistry
p-CREB: Phosphorylated cAMP-response element-binding protein
PDE: Phosphodiesterase
PKA: Protein kinase A
RAS: Renin-angiotensin system
TGF-β: Transforming growth factor-β
α-SMA: α-smooth muscle actin
