# Supplementary figures and images for: Dibutyryl-cAMP attenuates pulmonary fibrosis by blocking myofibroblast differentiation via PKA/CREB/CBP signaling in rats with silicosis
Source: Respir Res. 2017 Feb 21;18:38. doi: 10.1186/s12931-017-0523-z (PMC5320641; doi:10.1186/s12931-017-0523-z)

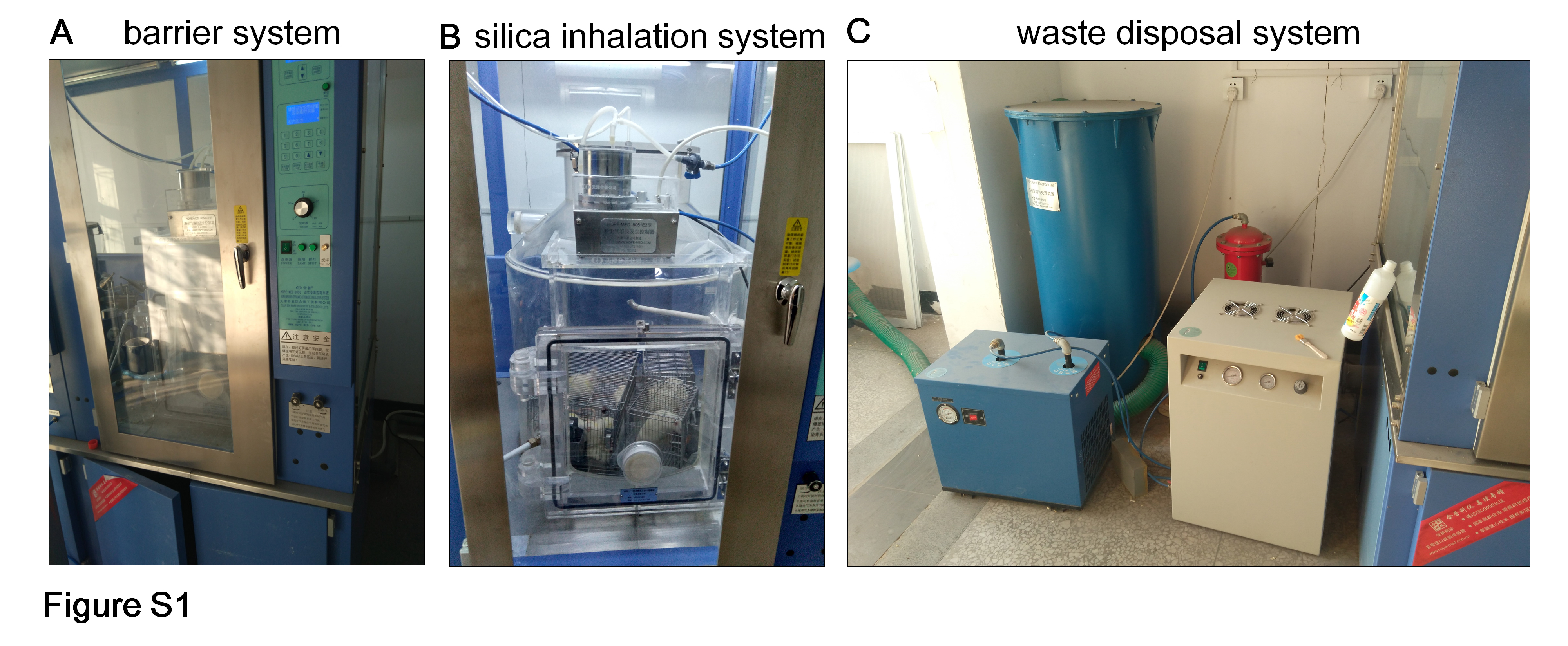

Supplement: Additional file 1: Figure S1. — The HOPE-MED 8050 exposure control apparatus. A) barrier system; B) silica inhalation system; C) waste disposal system (JPG 2785 kb) [file 12931_2017_523_MOESM1_ESM.jpg]

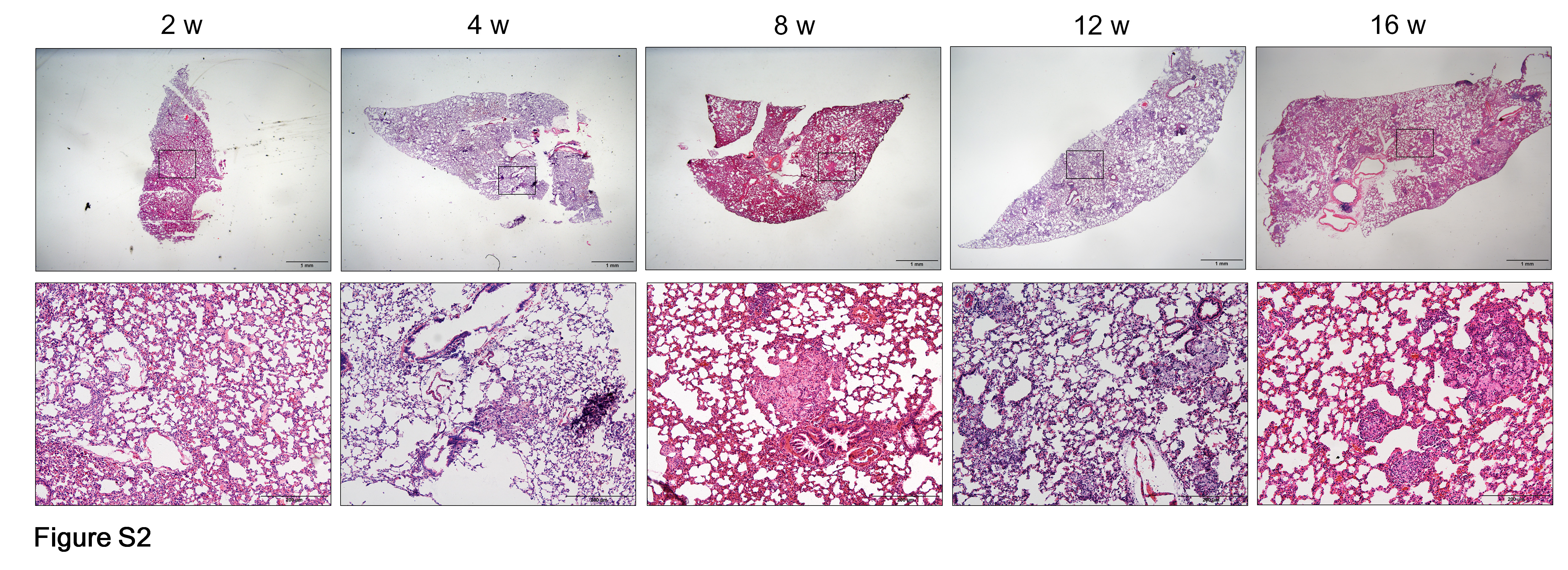

Supplement: Additional file 2: Figure S2. — The morphological observation of rat exposed to silica. (JPG 4540 kb) [file 12931_2017_523_MOESM2_ESM.jpg]
